# High-Resolution Rotation-Measuring System for MEMS Ultrasonic Motors Using Tunneling Magnetoresistance Sensors

**DOI:** 10.3390/mi15081028

**Published:** 2024-08-12

**Authors:** Jiangbo He, Qiuyue Feng, Yu Chen, Tianyu Yang, Xiaoshi Li, Wu Zhou

**Affiliations:** 1 School of Mechanical Engineering, Xihua University, Chengdu 610039, China; 3120200171275@stu.xhu.edu.cn; 2 Microsystem and Terahertz Research Center, China Academy of Engineering Physics, Chengdu 610299, China; yangtianyu_mtrc@caep.cn (T.Y.); lixiaoshi_mtrc@caep.cn (X.L.); 3 Institute of Electronic Engineering, China Academy of Engineering Physics, Mianyang 621900, China; 4 School of Mechanical and Electrical Engineering, University of Electronic Science and Technology of China, Chengdu 611731, China; zhouwu916@uestc.edu.cn

**Keywords:** MEMS, ultrasonic motor, rotation-measuring, tunneling magnetoresistance

## Abstract

This study proposes a high-resolution rotation-measuring system for miniaturized MEMS ultrasonic motors using tunneling magnetoresistance (TMR) sensors for the first time. Initially, the architecture and principle of the rotation-measuring system are described in detail. Then, the finite element simulation is implemented to determine the miniaturized permanent magnet’s residual magnetization, dimensions, and TMR sensor position. Finally, the experiments are implemented to evaluate the performance. Using calibration based on a high-precision servo motor, it is found that the relationship between the output and rotational angle is highly linear and immune to the rotor’s out-of-plane movement. Meanwhile, the angle-detecting resolution is higher than 0.1°. After the calibration, the continuous rotation of the MEMS ultrasonic motor is tested. It is found that the angle testing result varies with a period close to 360°, which indicates that the rotation-measuring system has successfully detected the motor’s rotation.

## 1. Introduction

The ultrasonic motor (USM) is an actuator that converts the stator’s vibration excited by the inverse piezoelectric effect into continuous rotor motion through friction [1]. The USM has wide applications in the fields of robotics [2,3], precision positioning systems [4], medical systems [5,6], and aerospace vehicles [7] due to advantages such as fast response, small size, and negligible electromagnetic interference.

In recent years, several studies have focused on miniaturized USM fabricated by wafer-scale microelectromechanical system (MEMS) techniques to reduce manufacturing cost, improve manufacturing precision, and enhance the capabilities of system-level integration. Rudy et al. proposed a MEMS USM made of a silicon layer and a thin lead zirconate titanate (PZT) film for the first time [8,9]. Zhou et al. used the saddle points of the modal shape to improve the quality factor of the MEMS USM [10,11]. Ran et al. established a three-dimensional contact model for the MEMS USM [12]. Zhao et al. proposed a MEMS USM with a thick PZT film to improve performance [13]. Hareesh et al. proposed a MEMS USM using the bulk PZT wafer to suppress the residual stress [14]. He et al. studied the traveling wave distortion in MEMS USM to improve performance [15].

Though the prototype of MEMS USM has been successfully fabricated, it is still a challenge to apply it in high-precision systems, such as precision positioning systems, because it is difficult to monitor the rotation precisely on a miniaturized scale. Optical and capacitive rotation-measuring systems are widely adopted for conventional macroscale motors. However, the optical rotation-measuring system is infeasible for the MEMS USM because the high-precision fabrication of a large number of gratings on a miniaturized scale is challenging, and a high-quality light source limits the size and cost [16]. In our previous study, a capacitive rotation-measuring system was established for MEMS USM [17]. However, the fabrication of the shielding layer for the parasitic capacitance is challenging and high-cost on a miniaturized scale [17]. Additionally, the rotor’s out-of-plane movement also induces a significant error because the out-of-plane movement changes the capacitive gap [17].

Compared to the optical and capacitive ones, the magnetic rotation-measuring system using tunneling magnetoresistance (TMR) sensors has the advantages of miniature size, high resolution, and low cost [18]. This kind of rotation-measuring system has been widely applied to macroscale rotation. For instance, Muna et al. explored the optimal magnetic field of the magnetic rotation-measuring system using TMR sensors by finite element analysis to realize the high resolution [19]. Bhaskarrao et al. proposed a TMR-based rotation-measuring system with a linearizing digital interface for shaft rotation [20,21]. Wang et al. presented TMR sensors from Multi Dimension Technology of China as a rotation-measuring element embedded into the permanent magnet synchronous motor [18,22].

Due to the advantage of miniature size, the TMR sensor has great potential for monitoring the rotation of miniaturized motors, such as the MEMS USM. However, there are no published reports so far. This study uses TMR sensors to establish a high-resolution rotation-measuring system for MEMS USM for the first time, which provides a feasible path to overcome the challenge of monitoring the rotation of miniaturized motors. Initially, the architecture and principle of the rotation-measuring system are described. Secondly, the finite element simulations are implemented to determine the miniaturized permanent magnet’s residual magnetization, dimensions, and TMR sensor position. Finally, the experiments are implemented, and the tested results are discussed.

## 2. Architecture and Principle of the Rotation-Measuring System

**Figure 1:**
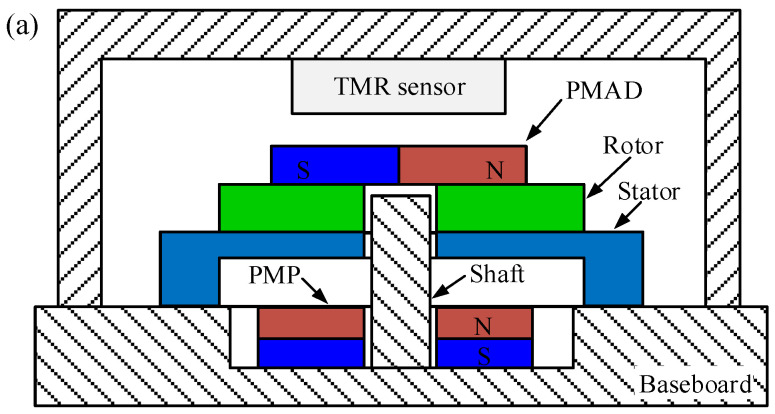

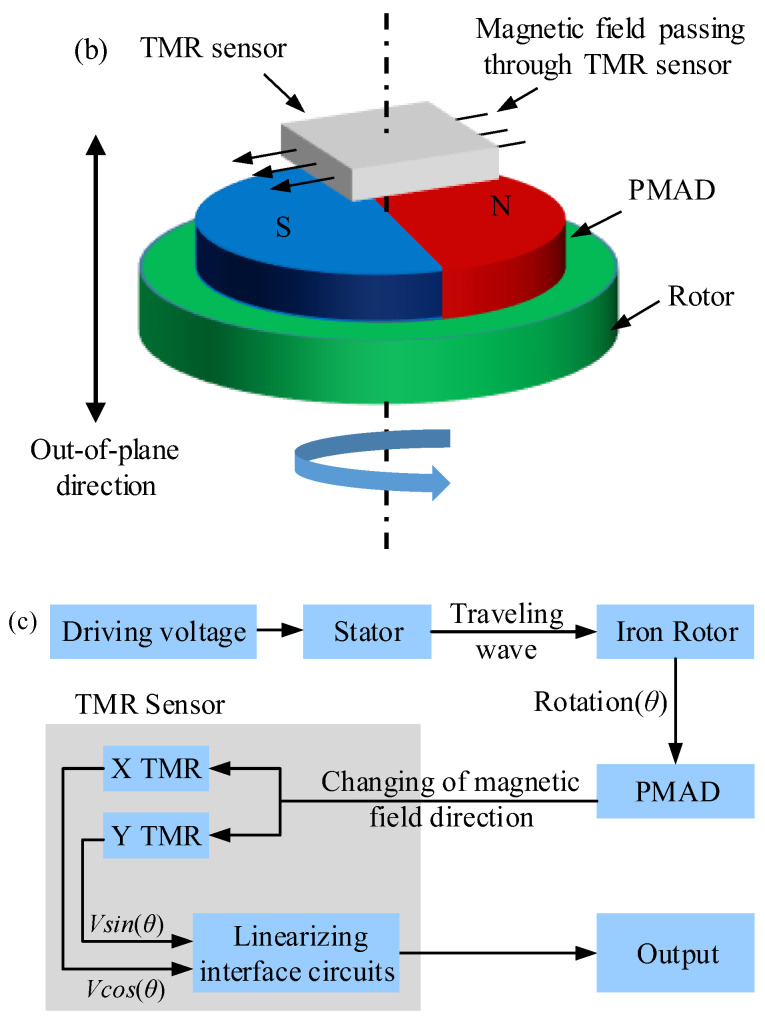
A schematic diagram for the rotation-measuring system: (**a**) geometric layout, (**b**) axonometric representation, (**c**) step-by-step block diagram describing the working principle.

A schematic diagram for the rotation-measuring system is shown in Figure 1. Firstly, the stator of the USM is mounted on the baseboard by adhesives. The miniaturized stator has a diameter as low as 4 mm. Then, an Iron rotor with a permanent magnet for angle detecting (PMAD) is placed on the stator in the out-of-plane direction, and an Aluminum shaft constrains its in-plane motions, as shown in Figure 1a. The PMAD is made of Neodymium Iron Boron and magnetized radially, as shown in Figure 1b. A TMR sensor is placed on the head of the PMAD, and its center is aligned with that of the PMAD. A preload for generating frictional driving force between the stator and rotor is necessary. In this work, the preload is produced by the attraction between the Iron rotor and a permanent magnet under the stator. The permanent magnet for preload (PMP) is vertical magnetization and also made of Neodymium Iron Boron.

**Figure 2:**
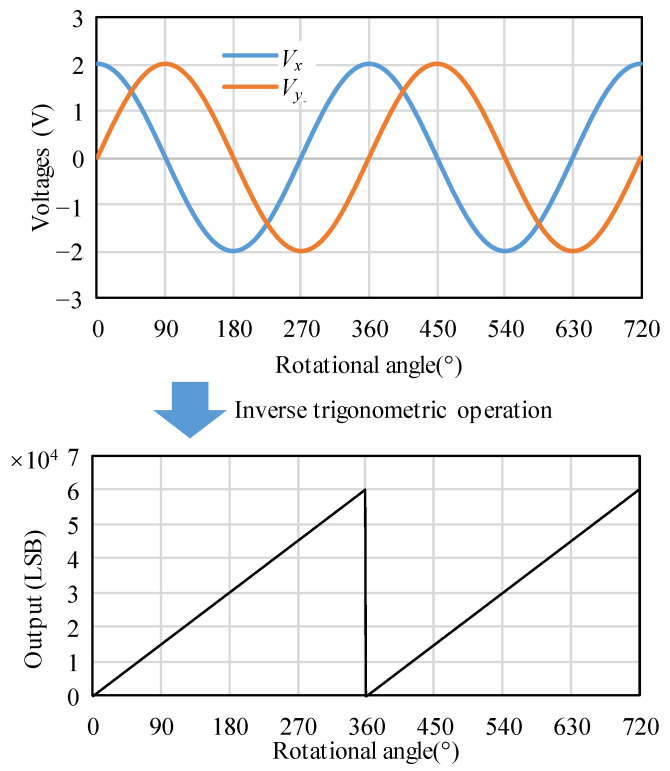
The Cos and Sin voltages are converted into the linear output by performing an inverse-trigonometric operation.

A step-by-step block diagram describing the working principle of the rotation-measuring system is shown in Figure 1c. When the driving voltages are applied, the traveling wave is excited on the stator by the inverse piezoelectric effect. Then, the traveling wave vibration is converted into continuous rotation of the rotor and PMAD through friction. The PMAD is magnetized radially, so the rotation of PMAD changes the direction of the magnetic field passing through the TMR sensor, as shown in Figure 1b. The X and Y elements of the TMR sensor change their resistance depending on the direction of the magnetic field, and the Wheatstone bridges transform the resistance variation into voltages with Cos and Sin waveforms [23]:

(1) $$\begin{array}{l} V_{x}=V_{p}cos\left(\theta \right)\\ V_{y}=V_{p}sin\left(\theta \right) \end{array}$$

where *V_x_* and *V_y_*, respectively, refer to the voltages produced by the X and Y elements of the TMR sensor, *V_p_* represents the peak value, and *θ* represents the rotational angle of the rotor. Finally, to yield the linear relationship between the output and rotational angle, the Cos and Sin voltages are input into the linearizing interface circuits to perform an inverse-trigonometric operation [24], as shown in Figure 2. Because the output of the inverse-trigonometric operation is 0°~360°, even as the rotational angle can continue to increase, the output will inevitably return to zero per 360° [24]. The turning point is at the zero-position where the output of the TMR sensor is zero, i.e., the magnetic direction makes *V_x_* maximum and *V_y_* zero [23].

## 3. Simulation of the Magnetic Field

The exact magnetic field in a compact space must be produced to satisfy the requirements of rotational measuring and preload. In this section, detailed finite element simulations of the magnetic field are implemented to determine the miniaturized permanent magnet’s residual magnetization, dimensions, and TMR sensor position. 

This study employs the commercial finite element software COMSOL Multiphysics 6.1 to simulate the magnetic field [25]. The diameters and thicknesses of the miniaturized permanent magnets and the rotor are listed in Table 1. The distance from the PMP to the rotor is 800 µm. These dimensions are determined to make the motor compact. Additionally, the PMAD’s diameter is smaller than the Iron rotor’s diameter to shield its effect on the magnetic attraction between the Iron rotor and PMP. Otherwise, a moment could be generated on the rotor to result in an inhomogeneous preload between the rotor and stator. The relative permeability of the stator, shaft, and baseboard is close to that of air, so they are all replaced by the air in the simulation model. The magnetization of the PMP is 400 kA/m. The PMAD’s residual magnetization and the TMR sensor’s position are parameters determined by the finite element simulations.

**Figure 3:**
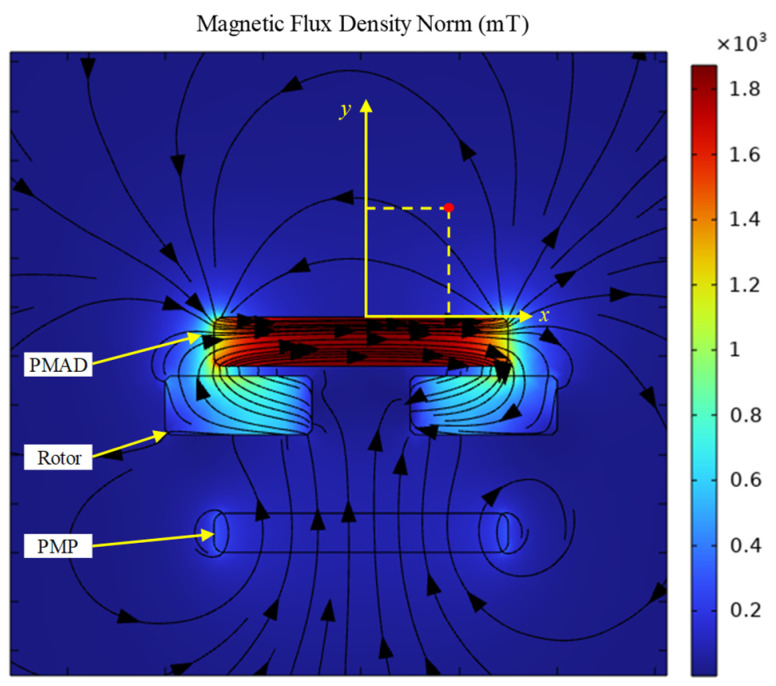
Finite element simulation results of the magnetic field. *x* and *y* represent the coordinates of points in the region above the PMAD.

**Figure 4:**
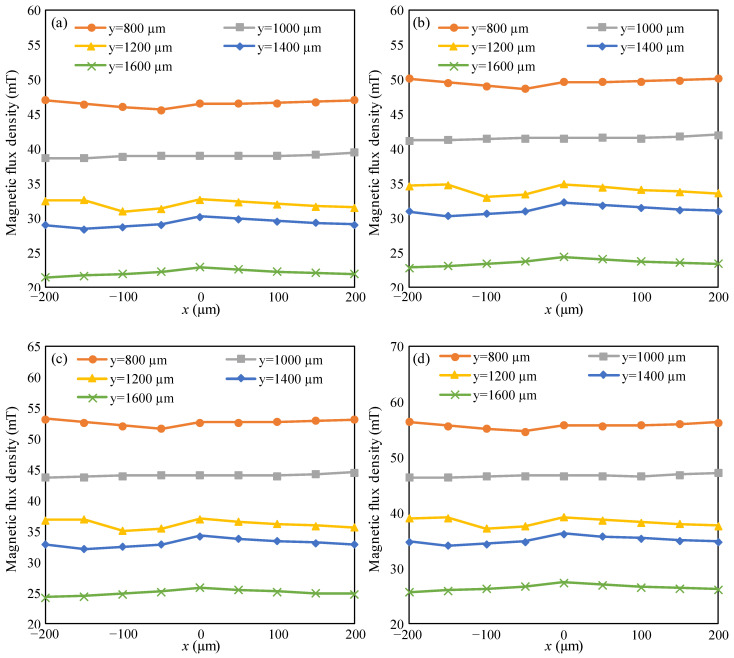
The variation of magnetic flux density in space: (**a**) residual magnetization of the PMAD is 1500 kA/m; (**b**) residual magnetization of the PMAD is 1600 kA/m; (**c**) residual magnetization of PMAD is 1700 kA/m; (**d**) residual magnetization of the PMAD is 1800 kA/m. *x* and *y* represent the coordinates of points in the region above the PMAD, as shown in Figure 3.

The simulation results are shown in Figure 3. The gap between the PMAD and rotor represents the space of adhesives. It can be seen from Figure 3 that the magnetic field lines above the PMAD correspond to the radial direction, which ensures that the TMR sensor can sense the rotation of the rotor. A Cartesian coordinate system is established to describe the variation of the magnetic flux density in space, as shown in Figure 3. The origin of the coordinate system is placed on the center of the top surface of the PMAD. The variation of the magnetic flux density is shown in Figure 4. It can be seen that the magnetic flux density increases with an increase in the residual magnetization of the PMAD and decreases with an increase in the vertical coordinate. However, the horizontal coordinate has a negligible effect on magnetic flux density because the magnetic field lines correspond with the radial direction. The minimum at about −50 μm may be induced by the effect of PMP on the magnetic field. If there is no PMP under the stator, the magnetic flux density must be symmetric with the middle line, and an extreme point of magnetic flux density must be on the middle line. However, when a PMP is mounted under the rotor, the symmetry of the magnetic field must be broken. As a result, the location for the extreme point could change from the middle to other points, such as the point that *x* = −50 μm.

The adopted TMR sensor in this paper comes from TDK Corporation and is labeled as TAD2141. TAD2141 is a digital sensor and has the advantages of high-accuracy angle sensing, miniature size, and low cost. The dimension of the TMR sensor is 5 mm × 4.4 mm × 1 mm, which is close to that of the MEMS USM. The magnetic flux density operating range for TAD2141 is from −20 mT to 80 mT [23]. In Figure 4c with the residual magnetization of 1700 kA/m, when the position of the TMR sensor varies from (0 mm, 0.8 mm) to (0 mm, 1.6 mm), the magnetic flux density varies from 55 mT to 25 mT. As a result, the requirement of the magnetic flux density for the angle detecting is satisfied.

**Figure 5:**
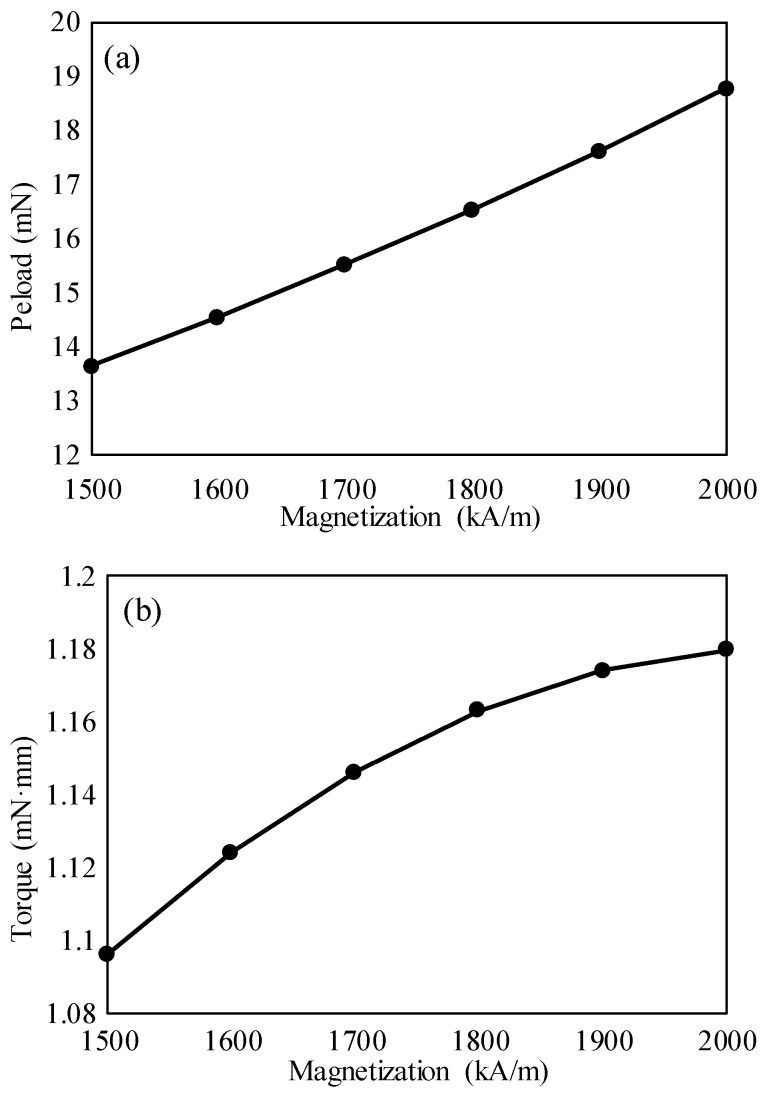
The dependences of preload and torque on the magnetization of the PMAD: (**a**) preload, (**b**) torque. The horizontal axis represents the magnetization of the PMAD.

According to the simulating results of the magnetic field, the preload provided by the magnetic attraction can be extracted by the built-in magnetic-force-calculation function of the COMSOL Multiphysics 6.1. The computed results of the preload are shown in Figure 5a. It can be seen that the preload increases with the increase of the residual magnetization of the PMAD. The reason is that the Iron rotor is magnetized more intensely when the residual magnetization of the PMAD is higher. According to our previous testing, a preload of about 16 mN is optimal for motor performance. When the residual magnetization is 1700 kA/m, the preload is close to 16 mN, so the requirement for the preload is also satisfied.

Besides the force, a small torque also exists on the rotor, as shown in Figure 5b. The small torque is induced by the interaction between the PMAD and PMP. According to the magnetic pole shown in Figure 1, the N-pole of the PMAD must be repulsed by the PMP, while the S-pole must be attracted by the PMP. However, it can be seen from Figure 5b that the torque is very small. For example, when the residual magnetization of the PMAD is 1700 kA/m, the resulting torque on the rotor is as low as 1.14 mN∙mm. Because the diameter of the rotor is 4 mm, the torque is equivalent to a pair of forces of 0.285 mN applied on the rotor, i.e., 1.14 mN∙mm = 2 × 0.285 mN × 2 mm. However, the preload applied on the rotor is 16 mN, which is much higher than 0.285 mN. Thus, this pair of forces has a very small effect on the preload. The reason is that the diameter of the PMAD is smaller than that of the Iron rotor, so interaction between the PMAD and PMP is shielded by the Iron rotor.

As a whole, the miniaturized PMAD with a residual magnetization of 1700 kA/m can satisfy both the requirements of the angle detecting and the preload.

## 4. Experimental Method

### 4.1. Fabrication and Assembly

**Figure 6:**
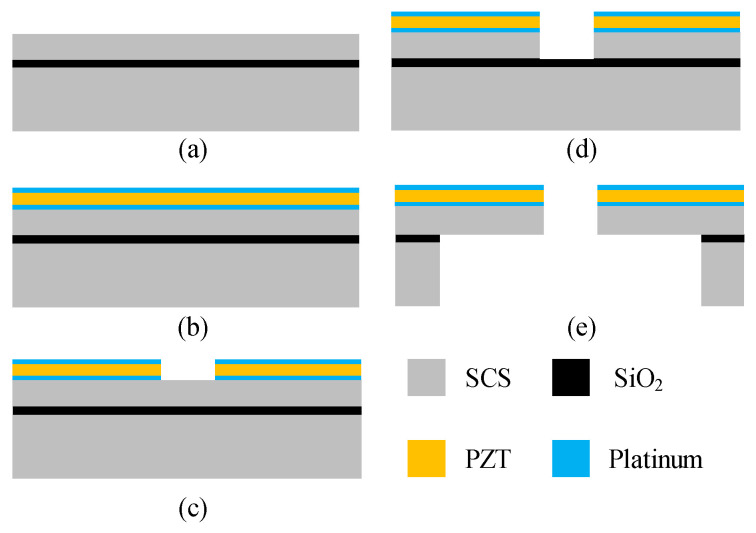
The fabrication process for the stator of the MEMS USM. SCS denotes the single-crystal silicon. Each step is detailed in the text. (**a**) Thinning the SOI wafer. (**b**) Sputtering a platinum layer for the first electrode. (**c**) Patterning the electrodes. (**d**) Shaping of the annular stator. (**e**) Releasing the whole structure.

The stator is fabricated by silicon-based wafer-level batch fabrication with film sputtering, as shown in Figure 6. At first, the device layer of an SOI wafer is thinned to 25 μm (Figure 6a). Then, a platinum layer is sputtered for the first electrode, followed by a 3 μm PZT film sputtered as the piezoelectric actuating layer; then, the second electrode is fabricated like the first one (Figure 6b). By wet etching and ion beam etching, the PZT and metal layers are etched to pattern the electrodes, the same as those in literature [10] (Figure 6c). Figure 6d shows the shaping of the annular stator by deep reactive ion etching (DRIE). Finally, the whole structure is released by DRIE on the backside (Figure 6e). 

**Figure 7:**
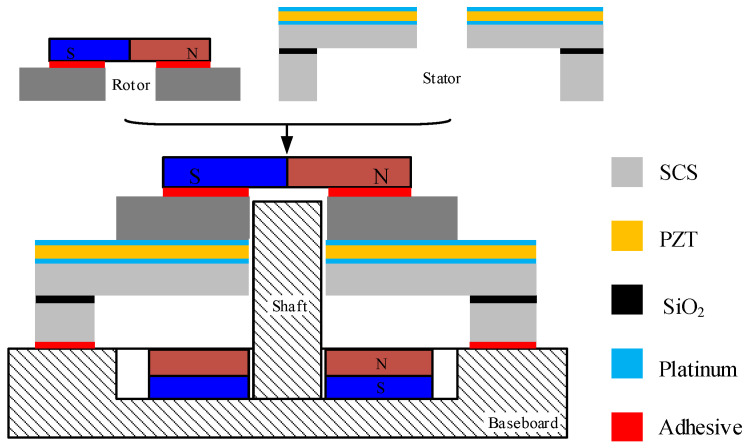
The rotor and stator are both assembled onto the baseboard to form the prototype of the MEMS USM.

**Figure 8:**
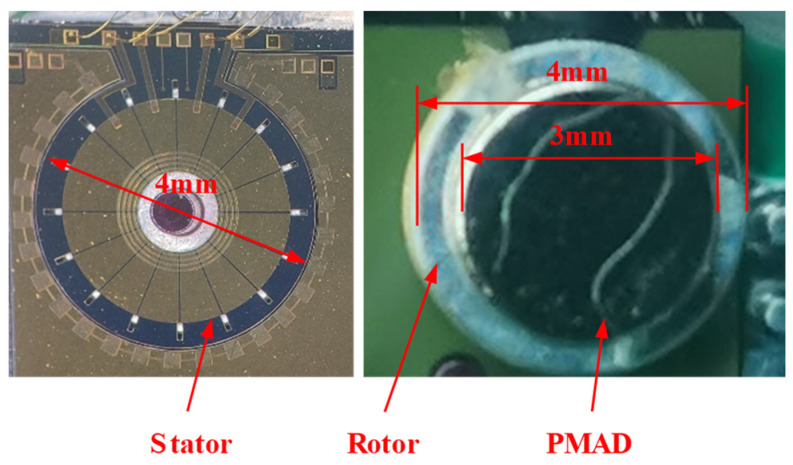
Two manufactured motors. The left one is without the rotor to display the stator, while the right one has the assembled rotor and PMAD.

After the release, the wafer is diced into the dies of the stator. Then, a die of the stator and a rotor are assembled into a complete motor, as shown in Figure 7, and the wires are bonded to connect the pads of the stator and the baseboard. The PMAD is bonded on the rotor using adhesive. This manual bonding may induce a slight alignment error between the rotor and the PMAD. In future, the alignment mark can be made on the rotor to suppress the alignment error. Two manufactured motors are shown in Figure 8. One of them is without the rotor to display the stator.

### 4.2. Testing Method

**Figure 9:**
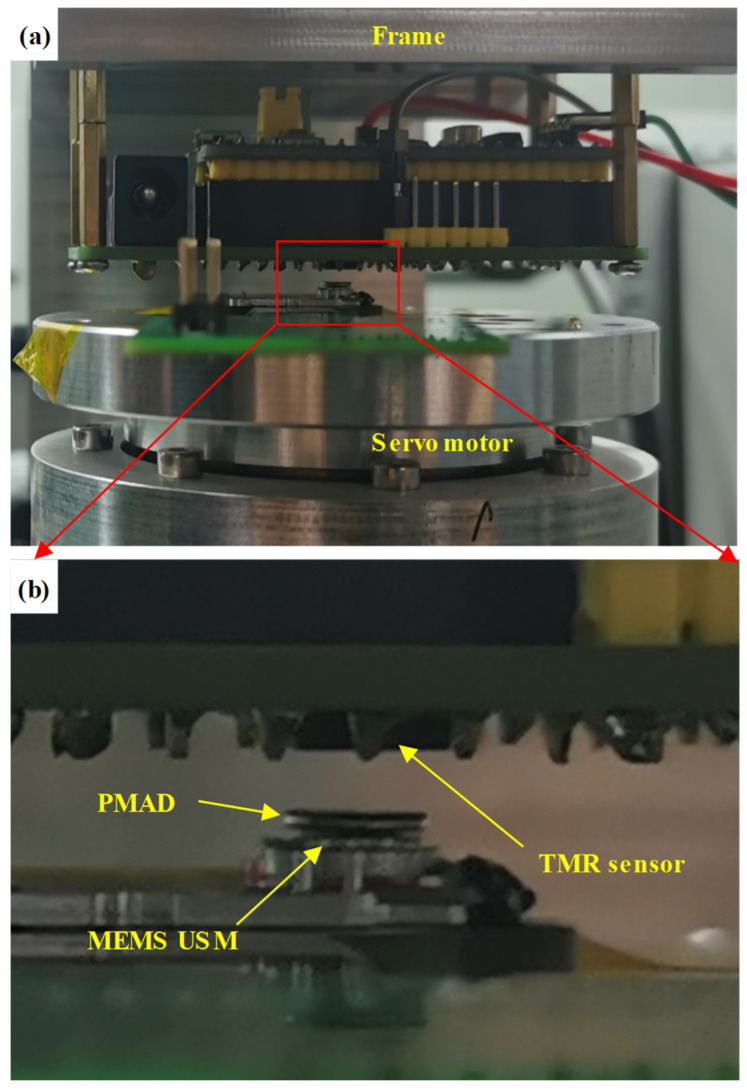
Testing architecture of the rotation-measuring system: (**a**) global layout of the measuring system, (**b**) zoom of the area enclosed by the red box in subgraph (**a**).

The testing architecture is shown in Figure 9. Its main components include a MEMS USM, a TMR sensor, a printed circuit board, a high-precision servo motor (HPSM), a positioning stage, etc. A customized PMAD with a residual magnetization of 1700 kA/m is mounted on the rotor of the MEMS USM by adhesives. The printed circuit board is designed to supply the power for the TMR sensor and transmit the digital output to a computer for data recording. The printed circuit board with the TMR sensor is mounted on a frame fixed on a high-precision positioning stage. This positioning stage can ensure that the center of the TMR sensor is aligned with that of the below PMAD and adjusts the distance from the TMR sensor to the PMAD. To calibrate the rotation-measuring system, the baseboard of the MEMS USM has adhered to the center of the HPSM, which can supply a high-precision rotational angle as low as 0.01°. Additionally, the high-precision encoder in the HPSM can output the rotational angle in real time.

In this paper, two testing modes are implemented, which are called the calibrating mode and continuous testing mode, respectively. 

In the calibrating mode, the MEMS USM itself is powered off, but the HPSM is powered on. Thus, the rotation of the PMAD is actuated by the HPSM in the calibrating mode, and then the output of the rotation-measuring system changes along with the rotation of the PMAD. Because the exact rotational angles are known from the high-precision encoder of the HPSM, the relationship between the output of the rotation-measuring system and the input rotational angle can be calibrated. 

In the continuous testing mode, the MEMS USM itself is powered on, but the HPSM is powered off. As a result, the driving voltage is input into the MEMS USM to make the PMAD rotate continuously. Then, the continuous rotation of the PMAD can be measured. The exact rotational angles are unknown in continuous testing mode. Thus, to obtain the rotational angle, the recorded outputs of the rotation-measuring system are converted into angles using the output–angle relationship acquired in the calibrating mode.

## 5. Experimental Results and Discussion

### 5.1. The Measuring Results of Calibrating Mode

**Figure 10:**
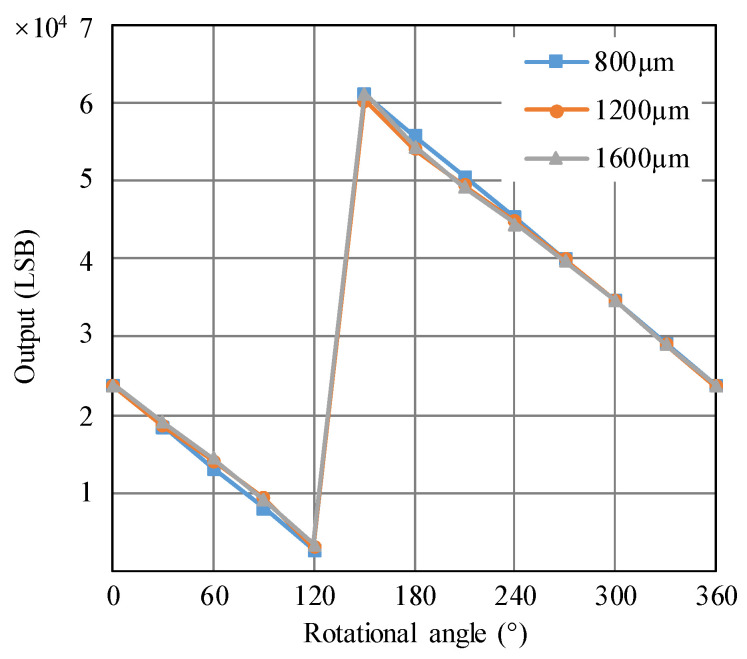
The relationship between the output of the rotation-measuring system and the rotational angle obtained in the calibrating mode. The horizontal axis represents the rotational angle relative to the initial position of the PMAD. The legend represents the distance from the PMAD to the TMR sensor. LSB is the abbreviation of the least significant bit, which is often employed as the output unit of digital sensors.

The HPSM actuates the PMAD to rotate clockwise with a step of 30°, and the outputs related to each step are recorded. The effect of the distance from the PMAD to the TMR sensor is also studied in this work. Thus, the measurements under three situations with distances of 800 µm, 1200 µm, and 1600 µm are implemented. The testing results are shown in Figure 10, where the horizontal axis represents the rotational angle relative to the initial position of the PMAD. The slight differences between the three testing results in Figure 10 may be induced by a non-uniform magnetic field because the magnetic flux density decreases with the increasing distance, as shown in Figure 4. The TMR sensor of TAD2141 from TDK of Japan integrated the linearizing interface circuits and sensing elements into a single die. Thus, the outputs of the rotation-measuring system are linearly dependent on the rotational angles. It can be seen that the output decreases with the increasing rotational angle, which is opposite to the law shown in Figure 2. This phenomenon is caused by the clockwise rotation. If the anticlockwise rotation is assumed to be positive, the output will increase with the increasing rotational angle, as with the law shown in Figure 2. Additionally, there is a turning point between 120° and 150°, which deviates from 360°, as shown in Figure 2. This deviation is caused by the misalignment between the pole of the PMA and the zero-position where the output of the TMR sensor is zero. The permanent magnet provided by the supplier does not have a label on the pole, so it is difficult to implement the alignment. If the angle from the initial position of the permanent magnet to the zero-position is between −240° and −210°, the turning point will fall into [120°, 150°].

In practice, the rotor has out-of-plane movement due to the adjustment of the preload, deformation induced by temperature variation, and out-of-plane vibration. In the previous study using a capacitive rotary encoder, the out-of-plane movement of the rotor caused a significant error because it changed the gap of the capacitor [17]. However, it can be seen from Figure 10 that the distance from the PMAD to the TMR sensor has a negligible effect on the output, though the step of distance is up to 400 µm. The reason is that the principle of the TMR sensor determines that it is only sensitive to the magnetic field direction. Because the distance variation induced by the rotor’s out-of-plane movement is much smaller than 400 µm, it can be concluded that the rotor’s out-of-plane movement has little effect on the output. Thus, the rotation-measuring system using the TMR sensor has the significant advantage of being immune to the rotor’s out-of-plane movement.

**Figure 11:**
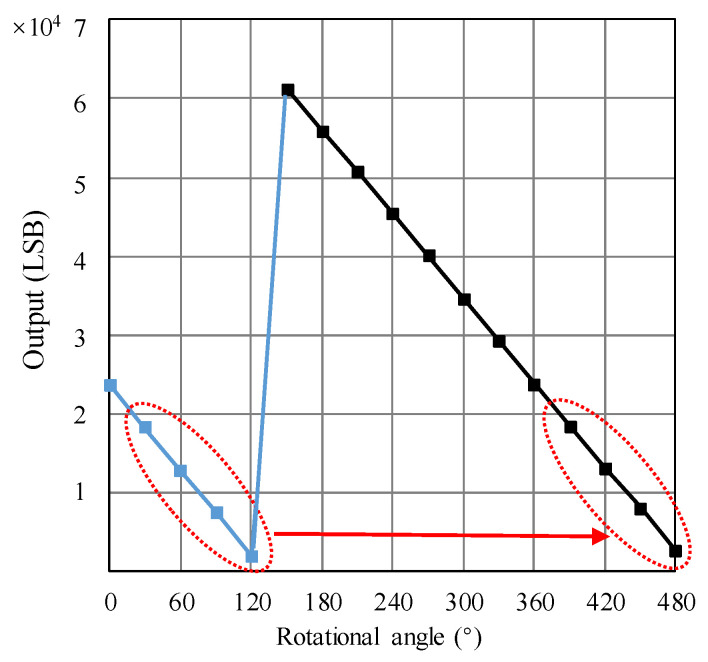
Data points of 30°, 60°, 90°, and 120° are moved to the equivalent 390°, 420°, 450°, and 480°. The data movement operation is implemented for the situation where the distance from the PMAD to the TMR sensor is 800 µm.

The linear regression is infeasible for the data shown in Figure 10 due to the turning point. Thus, the data points of 30°, 60°, 90°, and 120° are moved to the equivalent 390°, 420°, 450°, and 480° to avoid the jump, as shown in Figure 11. The data movement operation is implemented for the situation where the distance from the PMAD to the TMR sensor is 800 µm. As a result, when the angle varies from 150° to 480°, a continuous relationship between the angle and output is obtained. It can be seen from Figure 11 that the linearity of the relationship between the output and rational angle is very high. Implementing the linear regression of the data with the distance of 800 µm shown in Figure 11, the relationship between the output and rotational angle is expressed as

(2) O=−178.01x+87996

where *O* and *x* represent the output of the rotation-measuring system and rotational angle, respectively. In other words, *O* and *x* represent the *y* and *x* axes in Figure 11.

From Equation (2), the scale factor is 178.01 LSB/°. The root mean square error after the linear regression is 90.3 LSB. By dividing the root mean square error with the scale factor, the calculated nonlinear error is about 0.507°. The nonlinear error may be induced by the installation misalignment [26]. In the future, it will be valuable to study the correction method for the nonlinear error. The relationship between the output and rotational angle will be used in the continuous testing mode to evaluate the rotational angle.

**Figure 12:**
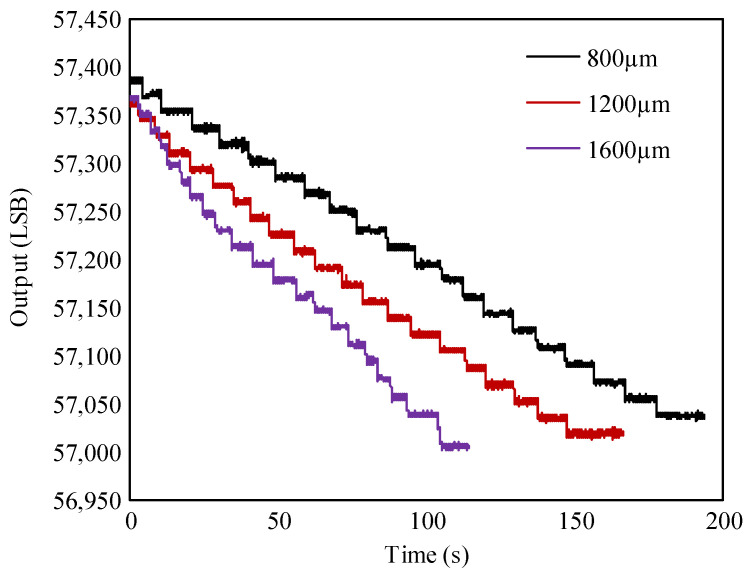
The output of the rotation-measuring system that the PMAD rotates with a step as small as 0.1°. The legend represents the distance from the PMAD to the TMR sensor.

To evaluate the detection resolution, the HPSM actuates the PMAD to rotate with a step as small as 0.1°, and 20 steps are completed. The testing results of the detection resolution are shown in Figure 12. The distance from the PMAD to the TMR sensor varies from 800 µm to 1600 µm. In each test, 20 steps are adopted to make the overall rotational angle approach 2°. Whatever the distance, it can be seen that the peak-to-peak noise is lower than the output variation induced by a step of 0.1° (about half). Thus, the detection resolution of the rotation-measuring system proposed in this study is higher than 0.1°. The detection resolution is mainly constrained by the resolution of the TMR sensor, which has a maximum of about 0.05° [23].

### 5.2. The Measuring Result of the Continuous Testing Mode

**Figure 13:**
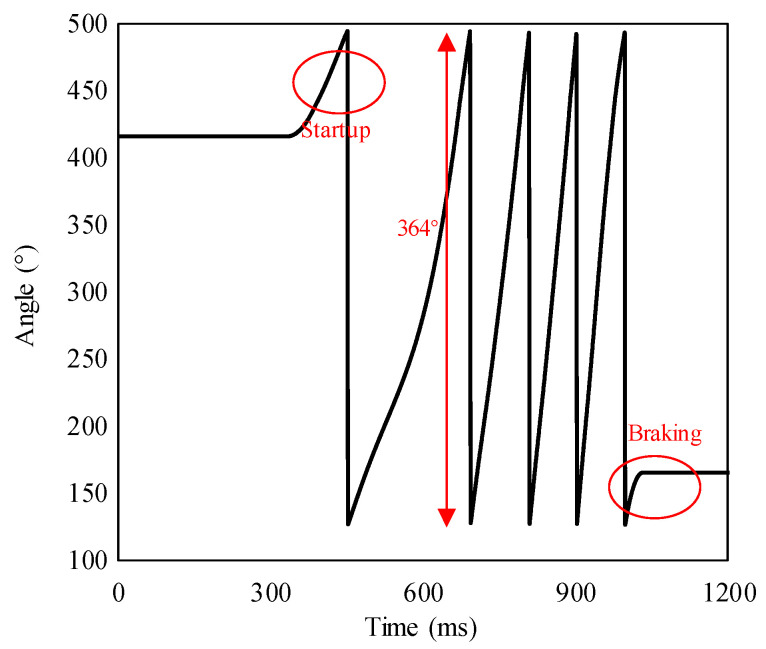
The rotation-measuring results where the MEMS USM actuates the PMAD into continuous rotation. The recorded outputs of the rotation-measuring system are converted into angles using the output–angle relationship acquired in the calibrating mode.

After calibration, the HPSM is powered off. Then, the driving voltage is input into the MEMS USM to rotate the PMAD continuously. The data sampling rate of the rotation-measuring system is 1000 Hz, and the distance from the PMAD to the TMR sensor is 800 µm. After the testing, the recorded continuous outputs of the rotation-measuring system are converted into angles using the output–angle relationship acquired in the calibrating mode, which is given in Equation (2). The converted result is shown in Figure 13. 

It can be seen that the angle does not vary initially because the MEMS USM is still asleep. After the MEMS USM is powered on, the rotor rotates and the angle starts to vary periodically. In Figure 13, the period at the steady state is about 93 ms, i.e., the rotational frequency is about 10.75 Hz. Thus, the rotational speed of the MEMS USM is about 645 r/min. Finally, when the MEMS USM is powered off, the rotor is braked by the sliding friction between the rotor and stator, and the angle falls stationary. The periodic variation of the angle indicates that the rotation-measuring system has successfully detected MEMS USM’s rotation. In each period, the angle initially increases with time and turns to the valley at a moment. In other words, the signal behaves like a sawtooth wave. Just like the results published in other reports [18,20], the sawtooth shape of the signal is regular because the TMR sensor employed in this paper has integrated the inverse-trigonometric operation. Thus, the relationship between the rotational angle and output behaves linearly. However, the rotation is periodic, so the signal must return to zero per 360°, as with the law shown in Figure 2.

**Figure 14:**
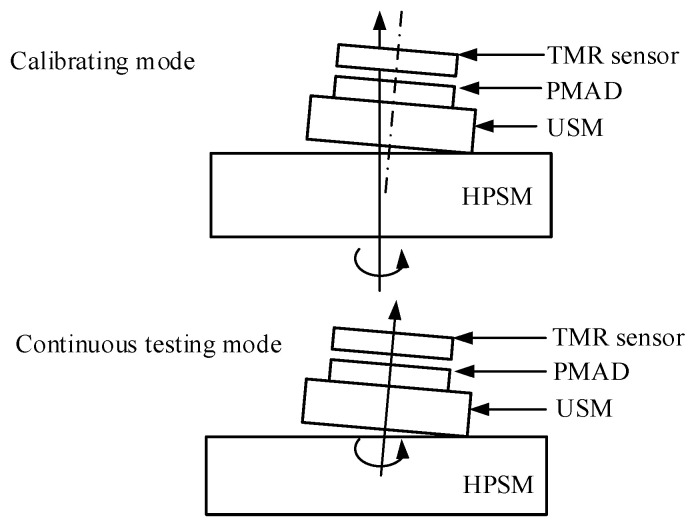
A schematic diagram for the assemble tilt error between the USM and HPSM. Due to the assembled tilt, the rotational angle actuated by the HPSM will be provided for the PMAD with loss.

The period of the angle is 364° and slightly higher than 360°. This deviation may be induced by the slight assemble tilt error between the USM and HPSM, as shown in Figure 14. In the calibrating mode, the MEMS USM is powered off, and the power-on HPSM actuates the PMAD’s rotation. However, the assemble tilt error makes the rotational angle around the axis of the PMAD lower than that actuated by the HPSM because of the vector characteristic of the rotation. On the contrary, in the continuous testing mode, the rotational angle actuated by the USM will be provided for the PMAD without loss because there is no tilt error between them. Thus, with the same rotational angle actuated by the HPSM and USM, the output from the calibrating mode will be lower than that from the continuous testing mode. As a result, when the continuous outputs of the rotation-measuring system are converted into angles using the output–angle relationship acquired in the calibrating mode, the obtained angles will be overestimated. In the future, a higher precision calibrating method, such as a high-speed camera [15], could be adopted to suppress the error.

Furthermore, it can be seen that the time consumption per loop is not constant. For example, the consuming time of the first loop is significantly longer than that of subsequent loops. This phenomenon is caused by the rotor’s rotation speed fluctuation, especially since the rotation speed is very unstable in the initial stage of rotation, which is induced by the traveling wave distortion of the stator [15]. The rotor’s rotation speed instability can generate significant positioning errors, which is why it is crucial to measure the rotation accurately. In the future, the servo control system based on the rotation-measuring system using the TMR sensor will be studied to realize accurate rotation.

## 6. Conclusions

This study proposed a rotation-measuring system for the millimeter-scale MEMS USM using TMR sensors for the first time. A PMAD with a radial magnetization of 1700 kA/m is attached to the rotor to satisfy the operating range of the TMR sensor and optimal preload for frictional driving. The calibration based on the HPSM shows that the relationship between the output and rotational angle is highly linear and immune to the rotor’s out-of-plane movement. After the calibration, the continuous rotation of the MEMS USM is tested. It is found that the angle derived from the rotation-measuring system’s output varies periodically, indicating that the measuring system has successfully detected the rotation of MEMS USM. The angle-detecting resolution of the rotation-measuring system is higher than 0.1° and is mainly constrained by the resolution of the TMR sensor. Overall, the rotation-measuring system has the advantages of high resolution, high linearity, and low cost.

In the future, it will be necessary to adopt a calibrating method with higher precision, such as using a high-speed camera, to suppress the calibration error. It is valuable to study the correction method for the nonlinear error. Additionally, it is valuable to study the servo control system based on the rotation-measuring system using the TMR sensor.

## Figures and Tables

**Table 1:** Dimensions used in the finite simulation model.

Component	Outer Diameter (µm)	Inner Diameter (µm)	Thickness (µm)
PMAD	3000	——	400
PMP	3000	——	400
Rotor	4000	1000	600

## Data Availability

Data are available upon request through a personal contact with the corresponding author at the email address: hejiangbo@foxmail.com.
